# Prenatal Volume in the Bilateral Superior Temporal Gyrus Associates With Children's Expressive Vocabulary at 24–36 Months

**DOI:** 10.1111/desc.70187

**Published:** 2026-04-11

**Authors:** Annika Werwach, Alex Tsompanidis, Luca Villa, Roger Tait, John Suckling, Topun Austin, Sarah Hampton, Carrie Allison, Rosemary Holt, Simon Baron‐Cohen, Gesa Schaadt

**Affiliations:** ^1^ Center for Lifespan Psychology Max Planck Institute for Human Development Berlin Germany; ^2^ Max Planck School of Cognition Leipzig Germany; ^3^ Autism Research Centre Department of Psychiatry University of Cambridge Cambridge UK; ^4^ Qynapse Paris France; ^5^ Cambridge Open Zettascale Lab University of Cambridge Cambridge UK; ^6^ The Rosie Hospital Cambride University Hospitals Foundation Trust Cambridge UK; ^7^ Department of Health Sciences University of York York UK; ^8^ University College London London UK; ^9^ Department of Education and Psychology Freie Universität Berlin Berlin Germany; ^10^ Department of Neuropsychology Max Planck Institute for Human Cognitive and Brain Sciences Leipzig Germany

**Keywords:** expressive vocabulary, language acquisition, prenatal brain development, structural MRI

## Abstract

Children's language development starts in utero, with language‐relevant brain areas starting to develop and differentiate during the second trimester of pregnancy. Postnatal development in language‐relevant brain areas such as the inferior frontal gyrus (IFG) and superior temporal gyrus (STG) has been shown to be related to language skills. In this study, as part of the Cambridge Human Imaging and Longitudinal Development (CHILD) project, prenatal structural characteristics of the IFG and STG (30^th^ – 33^rd^ GW) and their association with English children's language skills, obtained longitudinally at two postnatal assessment points (*n* = 24 and *n* = 25) was examined. Prenatal bilateral STG volume was found to be associated with expressive vocabulary 2–3 years after birth (*M* = 139.1 weeks), as measured by the Communicative Development Inventory (CDI). These results highlight the relevance of prenatal brain development for language acquisition after birth.

**Summary:**

- Postnatal structural characteristics of neural language network, including IFG and STG, are known to be related to language skills in children and adults
- Structural characteristics of IFG and STG were assessed prenatally in this study and related to language outcomes in early childhood
- Bilateral STG volume at birth predicts vocabulary scores 2–3 years later
- Findings support the importance of prenatal brain development for postnatal language acquisition

## 1 Introduction

Language development starts well before the infant is born, that is, in utero (Mahmoudzadeh et al. 2013; Moon et al. 2013). Although external speech signals are partially filtered by the womb, some speech sounds reach the fetus and are processed by their brain. Studies using behavioural, physiological, and neuroimaging data suggest that fetuses can react to speech stimuli (Fifer and Moon 1994), discriminate between different speech sounds (Mahmoudzadeh et al. 2013; Partanen et al. 2013; Shahidullah and Hepper 1994), process temporal variations of speech (Granier‐Deferre et al. 2011), and recognize familiar voices (Jardri et al. 2012; Kisilevsky et al. 2003, Kisilevsky et al. 2009). Thus, the neural structures underlying these processes ought to be, at least partially, already in place before birth. Indeed, the sylvian fissure can be observed from 20 gestational weeks (GW) on (Garel et al. 2001; Habas et al. 2012), with the formation of the inferior frontal gyrus (IFG) and the superior temporal gyrus (STG) being reported at around 24–25 GW (reviewed in Ghio et al. 2021). While these developmental periods are observed across fetuses, environmental and genetic factors can both influence timing and the extent of prenatal brain development during this period (Herzberg and Smyser 2024; Knickmeyer et al. 2014; Linderkamp and Linderkamp‐Skoruppa 2021).

Both IFG and STG are critical parts of the human neural language network (Friederici 2011) and play an important role in language acquisition (Skeide and Friederici 2016), with bilateral activations to speech information found in infants’ IFG and STG (Enge et al. 2020; Perani et al. 2011), while a left‐lateralized activation in these areas gradually emerges during early childhood (Friederici 2011, 2012). From three months on, interactions between bilateral STG and IFG support the emergence of language processing abilities and, with regard to expressive language, both regions are hypothesized to be relevant for infants’ ability to babble (Dehaene‐Lambertz et al. 2006; Skeide and Friederici 2016), a vital milestone of expressive language development (Oller et al. 1999; Werwach et al. 2021). Importantly, both IFG and STG have been shown to be associated with processes that are important for vocabulary acquisition: While IFG is involved in articulatory processes (Flinker et al. 2015; Hickok and Poeppel 2007), verbal working memory (Friederici 2011), and semantic processing (Enge et al. 2020; Friederici 2011, 2012), STG is involved in phoneme processing (Friederici 2011; Hickok and Poeppel 2007), semantic categorization (Skeide and Friederici 2016), as well as semantic‐syntactic integration (Friederici 2012). Further, both IFG and STG have been shown to play a role in mapping meaning to a new word (Mårtensson et al. 2012; Ye et al. 2011). Thus, both of these brain regions appear to be involved in overlapping but distinct processes that are important for vocabulary acquisition.

Given that (1) important prerequisites for later language skills (e.g., phoneme processing) rely on superior temporal and frontal regions (Garrido et al. 2009; Molholm et al. 2005), (2) these prerequisites are already in place in the first months after birth (Schaadt et al. 2023), and (3) structural characteristics such as volume or surface area of older children's IFG and STG are associated with concurrent and later language abilities (Gauger et al. 1997; Lu et al. 2006; Merz et al. 2020; Qi et al. 2021), it can be hypothesized that the structural characteristics of prenatal IFG and STG (i.e., parts of the neural language network) are already related to later language skills, setting the beginnings of postnatal language development. Supporting this hypothesis, a recent study found fetal temporal sulcus depth asymmetry to be related to expressive vocabulary skills later in childhood (Bartha‐Doering et al. 2023). The authors hypothesized an earlier structural and functional development of fetal language‐relevant brain areas to be associated with both less lateralized temporal sulcus depth and later language development, thus underlying the found relationship. However, the association between structural development of language‐relevant brain areas and later language skills has not yet been tested directly.

This study aimed to examine how structural characteristics of the prenatal neural language network, in particular the IFG and STG, are associated with early expressive vocabulary at 18 and 24–36 months after birth. Given prior evidence on the association of brain volume and language abilities (Gauger et al. 1997; Lu et al. 2006; Merz et al. 2020; Qi et al. 2021), we hypothesized prenatal IFG and STG volume to be positively related to later language development. Since bilateral involvement of the neural language network in language processing has been found in early infancy (e.g., Perani et al. 2011), we examined this association in both hemispheres exploratively without assuming a strong lateralization pattern.

## 2 Materials and Methods

### 2.1 Participants

Pregnant women were recruited at the Rosie Maternity Hospital, Cambridge, UK, as part of the Cambridge Human Imaging and Longitduinal Development (CHILD) project during their first (11–14 GW) or second prenatal monitoring appointment (18–21 GW), or by responding to adverts placed there. Only women with singleton pregnancies and who abstained from alcohol and smoking (self‐report) were included in this study. In addition, as part of an overarching project, the study was also advertised to mothers with an autism diagnosis or with a close family history of autism (partner or previous child) who were recruited as a ‘high likelihood’ group as previously described (Villa et al. 2021). MRI sessions were performed at the Rosie Maternity Hospital, Cambridge, UK. The study followed American Psychological Association standards in accordance with the declaration of Helsinki from 1964 (World Medical Association 2013), favourable ethical opinion was obtained (REC reference number: 12/EE/0393), and relevant approvals from the Research and Development Department of Cambridge University Hospitals NHS Foundation Trust. All mothers gave their written informed consent.

Of the combined cohort (both typical and high‐likelihood for autism), forty‐three women (*n* = 43) took part in prenatal magnet‐resonance‐imaging (MRI) scans of their child's brain between the 30^th^ and 33^rd^ gestational week (GW). Structural MRI data was not obtained from two fetuses due to motion artefacts, yielding a total sample size of *n* = 41 at prenatal assessment. From these 41 children, *n* = 27 and *n* = 25 mothers reported their child's expressive vocabulary via the MacArthur‐Bates Communicative Development Inventories—Short Form Level II (CDI), (Fenson et al. 2000) at 18 and 24–36 months, respectively. Children with a CDI score outside of the *Mean (M)* ± 2 *Standard Deviation (SD)* range (pre‐defined) were excluded from the analysis of the respective time point (*n* = 2 at 18 months, *n* = 1 at 24–36 months). These outlier scores correspond to the 90^th^ percentile or the 5^th^ percentile in the CDI‐short normative tables, respectively (Fenson et al. 2000). Excluded children fell within a ± 2 *SD* range of the sample mean concerning demographic variables (gestational age at scan, age at both language questionnaire assessments) and neural measures (left and right IFG and STG volume). Their sex distribution (2 female, 1 male) and background characteristics (White, monolingual households with mothers holding a university degree) were also comparable to the full sample.

Their exclusion resulted in a final sample size of *n* = 25 (at 18 months; 11 girls) and *n* = 24 (at 24–36 months; 13 girls) at the two assessment points. In this sample, vocabulary scores for both time points were available for *n* = 19 children. For *n* = 6 children, vocabulary scores were only available at 18 months, and for *n* = 5 children, vocabulary scores were only available at 24–36 months. See figure S1 for an illustration of participant inclusion and data loss at each stage of the study.

For descriptive statistics, only data from children of whom at least one CDI questionnaire was included after outlier exclusion is reported (*n* = 30, 14 girls). Importantly, for these 30 children, no maternal autism diagnosis was reported, while three children were reported to have a first‐degree relative with an autism diagnosis (parental report). Children were from predominantly White (87%), British families with middle to high socioeconomic backgrounds. Among mothers, 23% held a PhD, and 60% had a university degree (33% undergraduate, 27% postgraduate).

*Mean (M)* GW at the MRI scan was 31.61 weeks (*SD* = 1.19, *n* = 30). Mean age of children was *M* = 81.08 weeks (*SD* = 3.18, *n* = 25) at the 18‐month and *M* = 139.1 weeks (*SD* = 8.61, *n* = 23) at the 24–36‐month vocabulary assessment. All children were born full‐term (gestation week 37 or later, *M* = 39.63 weeks, *SD* = 1.23) with normal birth weight (above 2500 g, *M* = 3510.72 g, *SD* = 451.37). In the general sample, *n* = 13 kids were reported to have a second native language in addition to English (*n* = 9 in the 18‐month and *n* = 8 in the 24–36‐month‐group).

### 2.2 Procedure

Written informed consent was provided by all parents before each assessment at all time‐points. In the 30^th^ to 33^rd^ GW, an in utero fetal MRI scan was obtained. Expressive vocabulary was assessed 18 and 24–36 months after birth via parent report, using the MacArthur‐Bates Communicative Development Inventories—Short Form Level II (hence CDI) (Fenson et al. 2000). Parents were asked via email to complete the CDI online, together with other questionnaires on toddler behaviour and development, using an online platform hosted in secure servers of the University of Cambridge, and were compensated for their time and participation.

#### 2.2.1 MRI Acquisition

Scans were completed with a 1.5T MR450w Artist, GE Healthcare scanner at the Evelyn Perinatal Imaging Centre of the Rosie Hospital, Cambridge, UK. Total scan time for each participant was approximately 30 min. Structural images were acquired using the balanced steady‐state gradient echo sequence, Fast Imaging Employing Steady‐state Acquisition (FIESTA) with the following parameters: repetition time (TR) = 3.5 ms, echo time (TE) = 1.4 ms, flip angle (FA) = 55°, 172 slices with voxel size of 1.875 × 1.875 × 1mm^3^, field of view (FOV) = 256 × 256mm^2^ voxels. Anisotropic voxels were chosen to minimize scanning time and motion artifacts while maintaining sufficient signal‐to‐noise ratio, consistent with standard fetal MRI protocols (Ciceri et al. 2023; Glenn 2010).

All scans were reviewed by a neuroradiologist to ensure no incidental clinical findings warranted further clinical attention.

### 2.3 Structural MRI Pre‐Processing

Volume estimates were based on the STA31 template, which is part of a spatiotemporal MRI atlas for segmentation of early brain development (Gholipour et al. 2017). The STA31 parcellations are based on major cortical and subcortical landmarks rather than relying on gray/white matter contrast, making them suitable for indirect estimation of gray matter properties during this developmental period when tissue differentiation is limited. Multiple steps were taken to minimize registration and interpolation artifacts: First, brain images were manually reoriented using the Reorient tool in ITK‐SNAP (Yushkevich et al. 2006) to correct for mismatches between image orientation codes and the actual orientation of fetal brains that were caused by head movement in utero. The spatial origin was set to the anterior commissure fibre tract, to ensure precise co‐registration of the reoriented images. All images were manually skull‐stripped with the resulting region of interest (ROI) extending to the skull/cerebrospinal fluid interface and to the base of the cerebellum. A study‐specific template was then created from a sex balanced random participant sample using Advanced Normalization Tools (ANTs; Avants et al. 2009). Finally, spatial mappings from STA31‐to‐study‐specific template and subject‐to‐study‐specific template were combined into a single inversely symmetrical STA31‐to‐subject transformation to avoid multiple interpolation steps.

Due to the lack of a grey/white matter contrast in T1/T2‐weighted MRI images during this developmental period (Huang et al. 2006; Mori and Zhang 2006), direct segmentation of brain images into grey and white matter was not feasible. Thus, STA31 parcellations, which include parcellations of grey matter, white matter, and cerebrospinal fluid, were re‐sampled into acquisition space to indirectly estimate grey matter properties. The sum of combined re‐sampled STA31 parcellations was then used to estimate total brain volume from the structural images.

For the purpose of this study, volume estimates for left‐ and right‐hemisphere IFG and STG were used. Standard anatomical masks derived from the STA31 atlas parcellations (Gholipour et al. 2017) were used to define these regions in template space. The IFG parcel included the triangular, opercular, and orbital regions, while the STG parcel encompassed the superior temporal region. Masks were then transformed from template to each subject's native space to obtain regional grey matter estimates.

Left and right IFG and STG volumes were intracranial volume (ICV) normalized using the residual approach (see Mathalon et al. 1993; Wang et al. 2024). For this approach, regional volumes were regressed onto total ICV, and the residuals from this regression were used as subvolumes in the analyses.

### 2.4 Vocabulary Assessment

The CDI‐Short (Fenson et al. 2000) is a widely‐used parent‐report measure of early language development. It has been demonstrated to show high internal consistency (Cronbach's *α* > 0.90) and strong correlations with the long‐form CDI (*r* = 0.83 – 0.98, Fenson et al. 2000). Prior studies found the CDI‐Short to correlate with concurrent and later language abilities (Can et al. 2013; Pan et al. 2004).

For the purpose of this study, we operationalized children's expressive vocabulary via their raw score on the vocabulary checklist (range: 0–100), leaving the word combination question aside. Form A of the CDI‐Short was used at both assessment points. To examine whether the inclusion of bilingual children affected the results of this study, *t*‐tests were calculated comparing the CDI score of mono‐ and bilingual children from our sample at the 18‐ and the 24–36‐month assessment points separately. Since no significant differences in vocabulary scores were found at the 18‐ (*t*(15) = −0.72, *p* = 0.482) and the 24–36‐month assessment point (*t*(22) = −1.37, *p* = 0.182), bilingual children were included for further analysis.

Raw vocabulary scores were used for the analyses instead of age‐ and sex‐specific percentile scores, because normed percentile scores exist only for children younger than 31 months (Fenson et al. 2000). The effects of age and sex were controlled by adding the respective variables as covariates to the regression models.

### 2.5 Statistical Analysis

Statistical analyses were performed using R‐Studio version 3.6.3 (R Core Team 2020). First, *mean* and *SD* scores of raw fetal left‐ and right‐hemisphere STG and IFG volume and expressive vocabulary at 18 and 24–36 months were calculated. Further, correlations of ICV normalized fetal left‐ and right‐hemisphere STG and IFG volume with maternal age at time of MRI scan and gestational age at time of MRI scan were calculated and in case of a significant correlation, the respective variable was added as a covariate to the following analyses. To further control if there are potential influences of biological sex on fetal STG and IFG volume, independent *t*‐tests were computed comparing boys’ and girls’ fetal STG and IFG volume.

In a second step, zero‐order correlations (Pearson's *r*) between fetal left‐ and right‐hemisphere STG and IFG volume and expressive vocabulary at the age of 18 and 24–36 months were calculated.

To then evaluate the relation between fetal brain volume in language‐related regions and subsequent expressive vocabulary, multiple linear regression analyses were performed using ordinary least squares (OLS) estimation. Separate models were run for vocabulary scores at 18 months and at 24–36 months. Each model included ICV‐normalized IFG and STG subvolumes as predictors, together with their interaction with hemisphere as a categorical variable, to explore potential lateralization effects.

Age at the time of vocabulary assessment and biological sex were entered as covariates in all models. Maternal age and gestational age at time of MRI were included as additional covariates if they showed significant correlations with the regional brain volumes. Since neither maternal age nor gestational age at time of scanning were significantly associated with ICV‐corrected IFG or STG volume, these variables were not included in the final regression models.

The requirements for regression analyses, that is, homoscedasticity, linearity, and normal distribution of residuals, were all met based on plot inspection and lack of significant outliers. *P*‐values were corrected for multiple testing via the Bonferroni‐Holm correction (Holm 1979).

Overall model fit (indicated by *R^2^* and corrected *R^2^*) as well as individual regression coefficients of the two models were then examined. To confirm that our results were not impacted by the inclusion of three children (*n* = 3) with a first‐degree relative with an autism diagnosis (parental report), the analyses were repeated excluding these children. All three children were part of both the 18‐month‐ as well as the 24–36‐month‐sample. Thus, their exclusion lowered the sample size to *n* = 22 for analyses with vocabulary scores at 18 months as an outcome, and to *n* = 21 for analyses with vocabulary scores at 24–36 months as an outcome.

## 3 Results

### 3.1 Descriptive Statistics

All following scores and correlations were calculated after outlier exclusion (see 2.1). The mean CDI‐short vocabulary score was *M* = 18.72 (*SD* = 12.01) at 18 months and *M* = 84.67 (*SD* = 16.46) at 24–36 months. The mean fetal STG volume was *M* = 1085.43 voxels (*SD* = 192.65) in the left, and *M* = 1259.27 voxels (*SD* = 195.28) in the right hemisphere, while fetal IFG volume had a mean of *M* = 1874.43 voxels (*SD* = 445.95) in the left and *M* = 1865.8 voxels (*SD* = 406.95) in the right hemisphere. Please note that in the analysis, normalized subvolumes were used instead of total volumes.

There were no significant correlations between ICV normalized left and right fetal IFG and STG volume and gestational age or maternal age at time of MRI scan (see Table 1). There were also no significant differences between hemispheres for ICV normalized STG and IFG subvolume (STG: *t*(57.7) = 0.05, *p* = 0.959; IFG: *t*(48.6) = −0.07, *p* = 0.943). However, ICV normalized right IFG volume significantly differed between boys and girls (see Table 2). Consequently, only age at time of language assessment and sex were included as covariates in the regression models.

**TABLE 1:** Pearson's product‐moment correlations for ICV normalized left and right fetal STG and IFG volume with gestational age at time of MRI scan, total fetal brain volume and maternal age at scan.

		Gestational age at scan (years)	Maternal age at scan (years)
Fetal STG volume	Left	0.010	0.064
	Right	0.123	0.102
Fetal IFG volume	Left	0.246	−0.278
	Right	0.326^#^	0.272

^#^ *p* < 0.1.

* *p* < 0.05.

** *p* < 0.01.

*** *p* < 0.001.

**TABLE 2:** Descriptive statistics split by biological sex for fetal ICV normalized left‐ and right‐hemisphere STG and IFG volume. Comparison *t*‐tests reveal no significant difference between boys and girls in infants’ STG and IFG volume.

		*Mean (M)*		*SD*		Comparison by biological sex
		Girls	Boys	Girls	Boys	
Fetal STG volume	Left	58.4	−30.7	108.5	167.8	*t*(25.9) = 1.75, *p* = 0.092
	Right	46.0	−23.6	163.2	152.35	*t*(26.9) = 1.20, *p* = 0.240
Fetal IFG volume	Left	43.1	−17.26	335.5	406.5	*t*(27.9) = 0.45, *p* = 0.660
	Right	143.3	−94.3	182.1	214.6	*t*(28.0) = 3.28, ***p* = 0.003**

### 3.2 First‐Order Correlations: IFG and STG Volume and Expressive Vocabulary

Concerning the association between expressive vocabulary and ICV normalized fetal STG and IFG volume, there was no significant association between expressive vocabulary at 18 months and fetal right‐hemisphere STG volume, *r*(23) = 0.17, *p* = 0.425, left‐hemisphere STG volume, *r*(23) = 0.09, *p* = 0.683, right‐hemisphere IFG volume, *r*(23) = 0.28, *p* = 0.170, or left‐hemisphere IFG volume, *r*(23) = −0.10, *p* = 0.642. However, expressive vocabulary scores at 24–36 months significantly correlated with right‐hemisphere STG volume, *r*(22) = 0.55, *p* = 0.005, and left‐hemisphere STG volume, *r*(22) = 0.44, *p* = 0.031, as well as right IFG volume, *r*(22) = 0.45, *p* = 0.024. Left IFG volume was not significantly correlated with vocabulary scores at 24–36 months, *r*(22) = 0.19, *p* = 0.378.

### 3.3 Regression Analysis: IFG and STG Volume and Expressive Vocabulary

**FIGURE 1:**
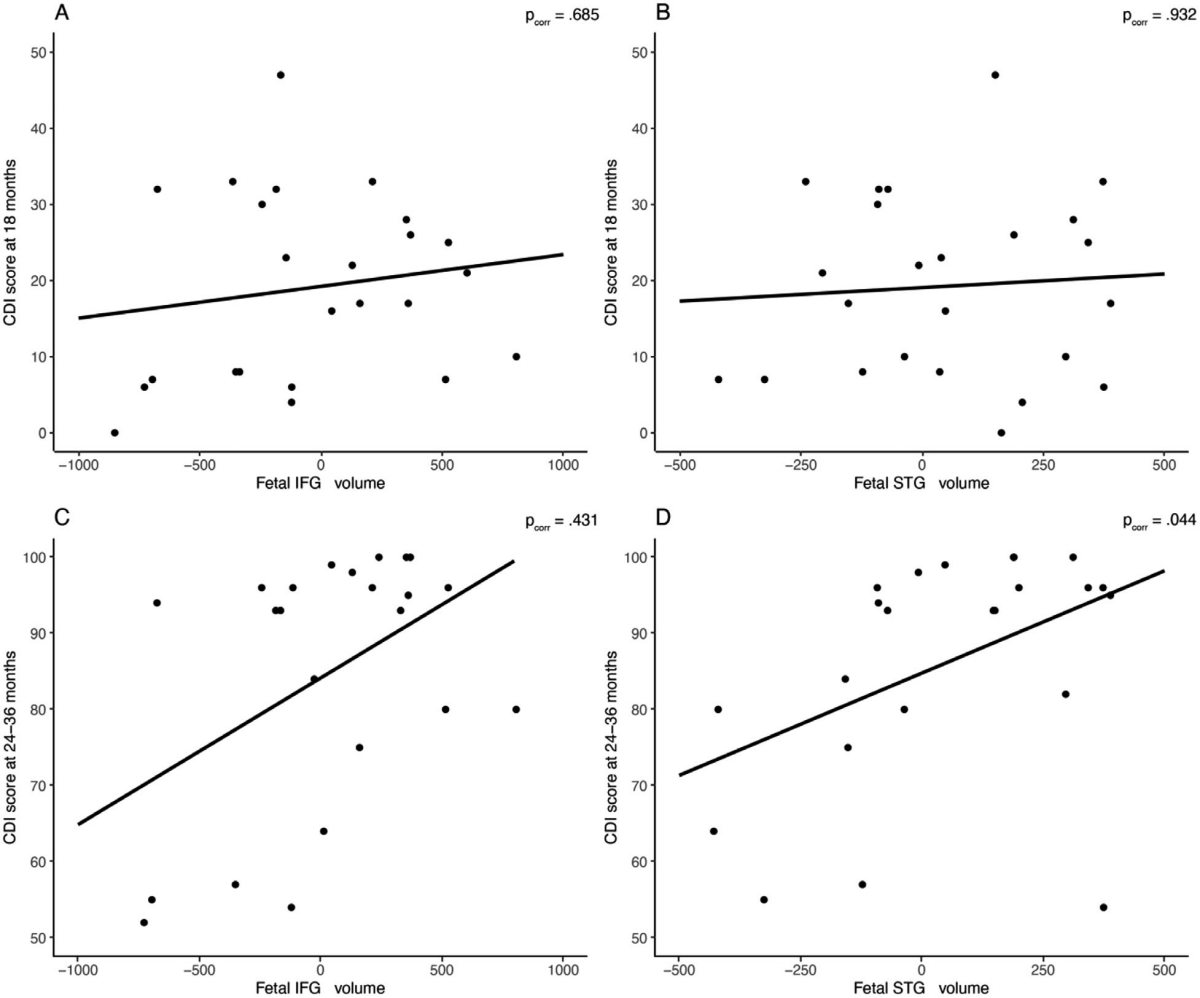
Regression of vocabulary scores at 18 and 24–36 months on bilateral ICV normalized fetal IFG and STG volume. *Note*: Regression plots of expressive vocabulary scores (assessed via the MacArthur‐Bates Communicative Development Inventories—Short Form Level II (CDI)) on fetal brain volume. **1A** Regression of expressive vocabulary scores at 18 months on bilateral ICV normalized fetal IFG volume (*n* = 25). **1B** Regression of expressive vocabulary score at 18 months on bilateral ICV normalized fetal STG volume (*n* = 25). **1C** Regression of expressive vocabulary score at 24–36 months on bilateral ICV normalized fetal IFG volume (*n* = 24). **1D** Regression of expressive vocabulary score at 24–36 months on bilateral ICV normalized fetal STG volume (*n* = 24). *P‐*values represent the significance of each individual predictor's regression weight (i.e., fetal STG and IFG volume) on expressive vocabulary at 18 months (1A, 1B) and 24 – 36 months (1C, 1D).

In the multiple regression analyses, expressive vocabulary scores at 18 months were not significantly predicted by ICV normalized fetal IFG or STG volume, *R^2^* = 0.233, *R_corr_^2^* = 0.118, *F*(7,42) = 1.93, *p* = 0.088, *p_corr_* = 0.088 (Table 3, see Figure 1A–B).

**TABLE 3:** Statistical parameters of the different predictors in the multiple linear regression models analyzing the association between ICV normalized fetal IFG and STG volume with vocabulary scores at 18 or 24–36 months, controlling for total brain volume and gestational age at time of MRI scan. *P*‐values are corrected for multiple comparisons using the Holm‐Bonferroni correction.

Model	Predictors	*b*	β	*t*(df)	*p*	*p_corr_*
Fetal IFG and STG volume as predictor of CDI score at 18 months	IFG volume	−0.002	−0.07	−0.41	0.685	0.685
	STG volume	0.001	0.02	0.09	0.932	0.932
	Hemisphere	−0.06	−0.002	−0.02	0.986	1.00
	Age at language assessment	0.87	0.23	1.55	0.129	0.129
	Sex	−11.23	−0.47	−3.13	**0.003** ^**^	**0.006** ^**^
	IFG volume x hemisphere	0.001	0.02	0.10	0.924	1.00
	STG volume x hemisphere	0.01	0.09	0.43	0.668	1.00
Fetal IFG and STG volume as predictor of CDI score at 24–36 months	IFG volume	0.01	0.18	1.26	0.216	0.431
	STG volume	0.06	0.50	2.39	**0.022** ^*^	**0.044** ^*^
	Hemisphere	−0.77	−0.02	−0.20	0.845	1.00
	Age at language assessment	0.84	0.44	3.13	**0.003** ^**^	**0.006** ^**^
	Sex	2.75	0.09	0.54	0.590	0.590
	IFG volume x hemisphere	0.002	0.02	0.12	0.906	1.00
	STG volume x hemisphere	0.001	0.01	0.07	0.948	1.00

^#^ *p* < 0.01.

* *p* < 0.05.

** p < 0.01.

The significant regression model predicting vocabulary scores at 24–36 months, *R^2^* = 0.438, *R_corr_^2^* = 0.339, *F*(7,40) = 4.45, *p* = 0.001, *p_corr_* = 0.002, showed ICV normalized STG volume to be significantly associated with expressive vocabulary scores 24–36 months later (see Table 3, see Figure 1 C–D), while IFG volume was not a significant predictor. Importantly, there was no significant interaction between STG or IFG volume and hemisphere, indicating both left and right STG volume were associated with later vocabulary scores.

The analysis excluding children with an increased likelihood of an autism diagnosis yielded consistent results (Suppl. Table S1): Both the regression models predicting vocabulary scores at 18 months, *R^2^* = 0.455, *R_corr_^2^* = 0.349, *F*(7,36) = 4.29, *p* = 0.002, *p_corr_* = 0.002 (see Table S1), and the regression model predicting vocabulary scores at 24–36 months reached statistical significance, *R^2^* = 0.491, *R_corr_^2^* = 0.386, *F*(7,34) = 4.69, *p* = 0.001, *p_corr_* = 0.002 (see Table S1). In the latter model, ICV normalized fetal STG volume again emerged as a significant predictor (corrected for multiple comparisons), while IFG volume did not significantly predict vocabulary scores at 24–36 months (see Table S1). There was no significant interaction between either STG or IFG volume and hemisphere, suggesting that both left and right‐hemisphere STG volume were associated with later vocabulary scores.

## 4 Discussion

The present study aimed to investigate if structural characteristics of the prenatal neural language network, specifically IFG and STG volume, are associated with language abilities (i.e., expressive vocabulary) after birth. Fetal brain scans were obtained between the 30^th^ to 33^rd^ week of gestation, and language skills were assessed via a parent‐report expressive vocabulary questionnaire 18 and 24–36 months after birth. We found bilateral fetal STG to significantly predict expressive vocabulary 24–36 months after birth, but not at 18 months.

Different structural and functional characteristics of the neural language network have been associated with language abilities both in children and in adults. The finding that structural characteristics of the prenatal neural language network already relate to language skills after birth suggest a continuity between prenatal and postnatal neural and behavioural language development. The stage of prenatal brain development during which MRI scans were obtained (30^th^ – 33^rd^ GW) is characterized by a remarkable surge in white matter growth (Jaimes et al. 2020), cortical folding (Tallinen et al. 2016), and the establishment of six‐layered lamination of the cortex (Mrzljak et al. 1988). Structural characteristics (e.g., volume) of the neural language network during this crucial period may set the basis for language developmental processes years later. Consistently, a recent study using a similar sample size compared to this study, reported that fetal temporal sulcus depth and its lateralization were associated with expressive language development 6–13 years of age postnatally and hypothesized an earlier development of the temporal cortex, including structural development, to underlie this association (Bartha‐Doering et al. 2023).

Interestingly, the investigated areas of the prenatal neural language network in the present study, that is, STG and IFG, were related differentially with later language skills, with the former being associated with subsequent expressive vocabulary and the latter showing no significant association. This suggests that prenatal variance in the superior temporal brain regions is more relevant for vocabulary acquisition earlier in life, as has been suggested in previous research on word learning in adults (Flöel et al. 2008; Mårtensson et al. 2012; Mei et al. 2008). Superior temporal regions have been shown to be directly involved in processes relevant for vocabulary acquisition, such as auditory and phoneme processing, lexical semantic categorization and lexical access and retrieval, whereas inferior frontal regions are suggested to receive information from the temporal cortex to then process these types of lexical and phonological information at a higher‐level (Friederici 2011; Skeide and Friederici 2016). Consequently, superior temporal regions may be more directly associated with vocabulary acquisition (see Skeide and Friederici 2016). Previous studies have found early temporal and only slow, repetition‐related frontal activation in response to speech in 3‐month‐old infants, indicating a sequential, hierarchical organization of activations to speech from superior temporal to inferior frontal regions (Dehaene‐Lambertz et al. 2002, Dehaene‐Lambertz et al. 2006). It has been proposed that this sequential organization may be indicative of different language‐related processing steps that operate on increasingly larger and more abstract parts of the speech signal, with earlier regions (i.e., superior temporal) processing smaller (e.g., words), and later regions (i.e., inferior frontal) processing bigger units of the speech signal (e.g., phrases, sentences) (Dehaene‐Lambertz et al. 2006). Thus, superior temporal regions may be more directly related to vocabulary acquisition (i.e., smaller units of the speech signal), while inferior frontal regions may contribute more strongly to more advanced or complex aspects of language development regarding, for example, phrases and sentences (e.g., syntactical acquisition).

Importantly, while adult studies consistently show associations between left‐hemisphere temporal activations with word learning (e.g., Friederici 2011, 2012), we found no effect of hemisphere in this study. Consequently, structural characteristics of the prenatal STG in both hemispheres appear to be important for later vocabulary acquisition. This may be due to the relevance of prosody for language development prenatally and in the early postnatal period (Höhle 2009; Kuhl 2004; Nallet and Gervain 2021), which is primarily processed in right hemisphere language regions (Gandour et al. 2004; Homae et al. 2006; Wartenburger et al. 2007). Since both prosodic information, processed in right hemisphere STG, as well as phonemic information, processed primarily in left hemisphere language regions (Friederici and Alter 2004), contribute to early word learning (Kuhl 2004), structural characteristics of both left and right prenatal STG may be equally important for vocabulary acquisition in the first years of life. This is supported by research showing bilateral activation in temporal regions in response to speech in newborns (Perani et al. 2011).

While we found a relationship between prenatal STG volume and expressive vocabulary at 24–26 months, there was no significant association between prenatal STG volume and vocabulary size at 18 months. This may be explained by the smaller expressive vocabulary and lower variability observed at the 18 month assessment point. Since around 18 months is the age when the expressive vocabulary spurt sets in (Bloom 2013; Kauschke and Hofmeister 2002) and a rapid increase in produced words is observed in the following months (Fenson et al. 1994), 24–36 months may be a more appropriate assessment point to investigate interindividual variability in expressive language skills and their predictors.

This study exclusively examined expressive vocabulary acquisition, which is a vital milestone of language development (Fenson et al. 1994). We focused on expressive vocabulary as it has been suggested to be more directly observable for parents (compared to receptive vocabulary) and, thus, to be more objectively measurable early in development, with fewer errors or reporting biases (see Houston‐Price et al. 2007; Tomasello and Mervis 1994). Receptive language development however, is equally important and should be evaluated in association with structural characteristics of the prenatal neural language network in future studies.

It should be noted that due to the small and primarily white sample from mainly middle to high socioeconomic backgrounds in this study, our findings warrant replication in larger, more representative and independent cohorts and ought to be treated as preliminary and exploratory. Attrition across the longitudinal assessments further reduced the number of participants with complete data at all time points, which is common in developmental studies due to missed follow‐up tests and participant withdrawal. Additionally, expressive vocabulary was assessed via a parental questionnaire, thus scores may be affected by reporting biases. The findings presented in this manuscript should therefore be validated using larger and more diverse samples and other measures of vocabulary size, such as direct testing in the lab, in future studies.

Additionally, due to the overarching goal of the CHILD project, which this study was part of, our sample included three children with an increased likelihood of an autism diagnosis later in life, indicated by the report of a first‐degree relative with an autism diagnosis. Since autism has been shown to be associated with alterations in language development (e.g., Eigsti et al. 2011), the inclusion of these children in our sample might have distorted our results. However, when excluding these three children from our analyses, the association between STG volume and later vocabulary remained significant. Moreover, vocabulary scores deviating more than two standard deviations from the mean were excluded in all analyses. This strongly suggests that the reported results are indicative of general associations between structural characteristics of the prenatal neural language network and later language skills, which are not specific to children with an increased likelihood of an autism diagnosis or a significant delay in language development.

In this study, a positive association was found between prenatal bilateral STG and expressive vocabulary 2–3 years after birth. In light of the relevance of early developmental trajectories for later language skills, this study shows that the prenatal development of the neural language network is related to postnatal language outcomes years later. By examining structural characteristics of the neural language network prenatally during a crucial period of brain development, this study shows that not only postnatal, but also prenatal volume in language‐related brain regions is linked to language development years later. These findings suggest continuity between prenatal and postnatal neural networks regarding language development, and could lead to further research on specific influencing factors that affect the development of the neural language network during pregnancy.

## Author Contributions

**Annika Werwach**: conceptualization, formal analysis, visualization, writing – original draft. **Alex Tsompanidis**: conceptualization, investigation, writing – review and editing, project administration, data curation. **Luca Villa**: formal analysis, methodology, writing – review and editing. **Roger Tait**: formal analysis, resources, writing – review and editing. **John Suckling**: resources, writing – review and editing. **Topun Austin**: resources, writing – review and editing. **Sarah Hampton**: project administration, investigation, writing – review and editing. **Carrie Allison**: conceptualization, writing – review and editing. **Rosemary Holt**: writing – review and editing, conceptualization, supervision, funding acquisition, project administration, resources. **Simon Baron‐cohen**: writing – review and editing, conceptualization, funding acquisition, resources, supervision. **Gesa Schaadt**: supervision, conceptualization, writing – review and editing, writing – original draft.

## Funding

S.B.C. received funding from the Wellcome Trust 214322∖Z∖18∖Z. For the purpose of Open Access, the author has applied a CC BY public copyright licence to any Author Accepted Manuscript version arising from this submission.

The results leading to this publication have received funding from the Innovative Medicines Initiative 2 Joint Undertaking under grant agreement No 777394 for the project AIMS‐2‐TRIALS. This Joint Undertaking receives support from the European Union's Horizon 2020 research and innovation programme and EFPIA and AUTISM SPEAKS, Autistica, SFARI. The funders had no role in the design of the study; in the collection, analyses, or interpretation of data; in the writing of the manuscript, or in the decision to publish the results.

S.B.C. also received funding from the Autism Centre of Excellence, SFARI, the Templeton World Charitable Fund and the MRC.

All research at the Department of Psychiatry in the University of Cambridge is supported by the NIHR Cambridge Biomedical Research Centre (BRC‐1215‐20014) and NIHR Applied Research Collaboration East of England.

The NIHR BRC is a partnership between Cambridge University Hospitals NHS Foundation Trust and the University of Cambridge, funded by the NIHR. The research was supported by the NIHR Collaboration for Leadership in Applied Health Research and Care East of England at Cambridgeshire and Peterborough NHS Foundation Trust. T.A. is supported by the NIHR BRC and by the NIHR HealthTech Research Centre for Brain Injury.

Any views expressed are those of the author(s) and not necessarily those the NHS, NIHR, Department of Health and Social Care, IH‐JU2 or other funders.

## Ethics Statement

The study followed American Psychological Association standards in accordance with the declaration of Helsinki from 1964 (World Medical Association 2013), favourable ethical opinion was obtained (REC reference number: 12/EE/0393), and relevant approvals from the Research and Development Department of Cambridge University Hospitals NHS Foundation Trust.

## Conflicts of Interest

The authors declare no competing interests.

## Supporting information




**Supplementary Information**: desc70187‐sup‐0001‐SupMat.docx

## Data Availability

The analysis script can be found under https://github.com/annikawerwach/CHILD‐volume‐vocab/tree/main/materials. Access to raw data is restricted by ethics regulations and participant consent.
