# Supplementary material for: Prenatal Volume in the Bilateral Superior Temporal Gyrus Associates With Children's Expressive Vocabulary at 24–36 Months
Source: Dev Sci. 2026 Apr 11;29:e70187. doi: 10.1111/desc.70187 (PMC13069930; doi:10.1111/desc.70187)
Supplement: Supplementary file 1 — Supplementary Information: desc70187‐sup‐0001‐SupMat.docx [file DESC-29-e70187-s001.docx]

**Supplementary Material for Manuscript “Prenatal volume in the bilateral superior temporal gyrus associates with children’s expressive vocabulary at 24-36 months”**

**Figure S1.**

*Participant flow chart illustrating the number of included participants at each stage of the study.*

**
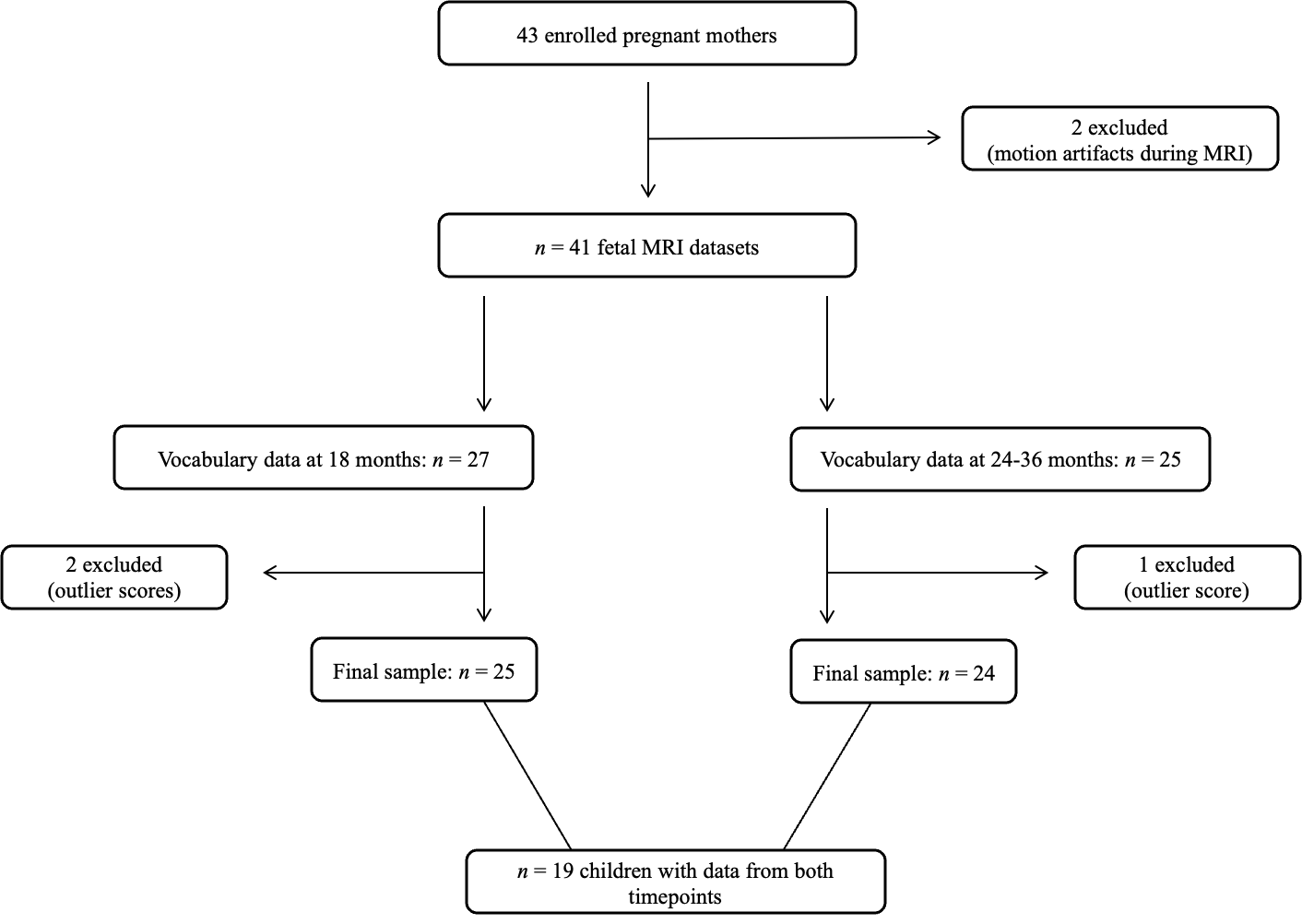
**

*Note*. Flow chart of participant inclusion and data loss at each stage of the present study. Reasons for exclusion (motion artifacts, language scores > ±2 SD from sample mean) are indicated. The final analytical sample consisted of 25 children at the 18-month assessment point and 24 children at the 24-36-month-assessment point. 19 children contributed data at both time points.

**Table S1**

*Statistical parameters of the different predictors in the multiple linear regression models analysing the association between fetal IFG or STG volume with vocabulary scores at 18 or 24-36 months, controlling for total brain volume and gestational age at time of MRI scan, excluding the children with a first-degree relative with an autism diagnosis. P-values are corrected for multiple comparisons using the Holm-Bonferroni correction.*

| Model | Predictors | b | β | *t*(df) | *p* | *P_corr_* |
| --- | --- | --- | --- | --- | --- | --- |
| Fetal IFG and STG volume as predictor of CDI score at 18 months | IFG volume | -0.01 | -0.18 | -1.21 | .235 | .470 |
|  | STG volume | -0.02 | -0.23 | -1.12 | .268 | .268 |
|  | Hemisphere | -0.78 | -0.03 | -0.27 | .790 | 1.00 |
|  | Age at language assessment | 1.30 | 0.29 | 2.21 | **.034*** | **.067^#^** |
|  | Sex | -15.02 | -0.64 | -4.68 | **<.001**** | **<.001**** |
|  | IFG volume x hemisphere | 0.003 | 0.05 | 0.31 | .755 | .721 |
|  | STG volume x hemisphere | 0.02 | 0.19 | 0.93 | .361 | 1.00 |
| Fetal IFG and STG volume as predictor of CDI score at 24-36 months | IFG volume | 0.003 | 0.08 | 0.56 | .583 | .583 |
|  | STG volume | 0.06 | 0.57 | 2.52 | **.017*** | **.033*** |
|  | Hemisphere | -0.74 | -0.03 | -0.21 | .834 | 1.00 |
|  | Age at language assessment | 0.49 | 0.31 | 2.00 | **.054^#^** | **.067^#^** |
|  | Sex | -2.27 | -0.08 | -0.49 | .631 | .631 |
|  | IFG volume x hemisphere | -0.01 | -0.07 | -0.36 | .720 | 1.00 |
|  | STG volume x hemisphere | 0.01 | 0.04 | 0.19 | .850 | .850 |

^#^ *p* < .1; * *p* < .05; ** p < .01
